# Extracts of centipede *Scolopendra subspinipes mutilans* induce cell cycle arrest and apoptosis in A375 human melanoma cells

**DOI:** 10.3892/ol.2014.2139

**Published:** 2014-05-12

**Authors:** WEINA MA, RUI LIU, JUNPENG QI, YANMIN ZHANG

**Affiliations:** School of Pharmacy, Health Science Center, Xi’an Jiaotong University, Xi’an, Shaanxi 710061, P.R. China

**Keywords:** centipede *Scolopendra subspinipes mutilans*, cell growth, cell cycle, apoptosis

## Abstract

Extracts from the centipede *Scolopendra* genus, have been used in traditional medicine for the treatment of various diseases and have been found to exhibit anticancer activity in tumor cells. To investigate the potential and associated antitumor mechanism of alcohol extracts of the centipede *Scolopendra subspinipes mutilans* (AECS), cell viability, cell cycle and cell apoptosis were studied and the results revealed that AECS inhibits A375 cell proliferation in a dose- and time-dependent manner. In addition, AECS was found to arrest the cell cycle of A375 cells at the S phase, which was accompanied by a marked increase in the protein levels of cyclin E and a decrease in the protein levels of cyclin D1. In a cell culture system, AECS markedly induced the apoptosis of A375 cells, which was closely associated with the effects on the Bcl-2 family, whereby decreased Bcl-2 and increased Bak, Bax and Bad expression levels were observed. The underlying mechanism of AECS inhibiting A375 cell proliferation was associated with the induction of cell cycle arrest and apoptosis, indicating that AECS may present as a potential therapeutic agent for administration in human melanoma cancer intervention.

## Introduction

The class, Chilopoda contains ~3,500 described species of centipedes, distributed among five living orders, which are predators characterized by a dorsoventrally flattened body bearing ≥15 pairs of legs; one pair per trunk segment (1). Centipedes are predators that use venom to primarily arrest or subdue their prey and have been used, particularly *Scolopendra subspinipes mutilans* (*S. subspinipes mutilans)*, in Eastern medicine to treat a variety of conditions, including spasms, childhood convulsions, seizures, poisonous nodules and diphtheria (2,3). Venom is a key element in the predatory behavior of centipedes, however, analysis of such has only been performed on large scolopendromorphs known to have significant importance in medical treatment. The composition and structure of centipede venom remains largely unknown, however, previous studies have revealed that serotonin, histamine, lipids, polysaccharides and polypeptides have been identified in the crude extracts of centipede venom glands (4,5). The ethanol extract of *S. subspinipes mutilans* has also been reported to exhibit marked cytotoxic activity against human cancer cells (2).

Cell cycle deregulation, resulting in uncontrolled cell proliferation, is one of the most common alterations that occurs during tumor development. Furthermore, cell cycle arrest is considered to be an effective strategy for eliminating cancer cells (6). Two major checkpoints, one at the G1/S transition and one at the G2/M transition, regulate the cell cycle and, therefore, the modulated expression of cell cycle regulatory molecules on antiproliferation or apoptosis has been investigated in numerous cell types (7). A general critical event associated with DNA damage is the activation of cell cycle checkpoints, and cyclins and cyclin-dependent kinases (cdks) are evolutionarily conserved proteins that are essential for cell cycle control (8). Distinct pairs of cyclins and cdks regulate the progression through the various stages of the cell cycle; cdk activity is regulated by cyclins, which bind to and activate cdks (9). The present study investigated whether AECS-induced antiproliferation or apoptosis are associated with an uncontrolled cell cycle.

Apoptosis, which effectively reduces the size of tumors and prevents further tumor growth, is a predominant type of cell death, which is characterized by a series of stereotypic molecular features, including the expression and translocation of the Bcl-2 family proteins, release of cytochrome *c* and activation of caspases (7). The human Bcl-2 homologs comprise the major apoptosis regulatory gene family and the Bcl-2 family of proteins may be divided into two groups; apoptosis suppressors (including Bcl-2, Bcl-xl and Mcl-1) and apoptosis activators (including Bax, Bak, Bid and Bad) (10). A variety of theories regarding the mechanism of action of the Bcl-2 family have been presented and the accumulating data indicates that these proteins function at numerous stages of the signaling cascade, which results in apoptosis (11).

Therefore, the present study evaluated the antitumor activity of the alcohol extracts of the centipede *S. subspinipes mutilans* (AECS) and investigated the mechanism of AECS inducing cell cycle arrest and apoptosis, for use in cancer treatment.

## Materials and methods

### Chemicals and reagents

Adult specimens of the centipede *S. subspinipes mutilans* were purchased from LaoBaiXing Pharmacy (Xi’an, China) and identification of the specimens was performed at the Pharmacology Laboratory, Xi’an Jiaotong University (Xi’an, China) where a voucher specimen was deposited. RPMI-1640 medium, dimethyl sulfoxide (DMSO) and trypsin were purchased from Sigma-Aldrich (St. Louis, MO, USA) and 3-(4, 5-dimethylthiazol-2-yl)-2.5-diphenyl-2H-tetrazolium bromide (MTT) was purchased from Nanjing Sunshine Biotechnology Ltd. (Nanjing, China). The Annexin V-fluorescein isothiocyanate (FITC) apoptosis detection and Hoechst 33258 staining kits were purchased from Beyotime Institute of Biotechnology (Shanghai, China). RNase and propidium iodide (PI) were purchased from Sigma-Aldrich, and protease inhibitor and phosphatase inhibitor cocktails were purchased from Roche Technology (Basel, Switzerland). The anti-CDC2, -CCNB1, -cyclin D1, -cyclin E, -Bad, -Bak, -Bax, -Mcl-1 and -Bcl-2 antibodies were purchased from Cell Signaling Technology, Inc. (Danvers, MA, USA). The rabbit anti-GAPDH was purchased from Santa Cruz Biotechnology, Inc. (Santa Cruz, CA, USA) and rabbit anti-mouse immunoglobulin G, bicinchoninic acid protein assay reagent kit and SuperSignal^®^ West Pico Chemiluminescent substrate were all purchased from Pierce Biotechnology, Inc (Rockford, IL, USA).

### Cell culture

Human A375 melanoma cells, obtained from the Shanghai Institute of Cell Biology in the Chinese Academy of Sciences, were maintained in RPMI-1640 and supplemented with 10% (v/v) fetal bovine serum (FBS) at 37°C in a 5% CO_2_ incubator with saturated humidity.

*Centipede S.* genus extract. The centipede *S. subspinipes mutilans* was shattered into a fine powder and 50 g of the centipede *S. subspinipes mutilans* was decocted in 1,500 ml ethanol solution [3/2 (v/v); ethanol/water] for 1 h. The solution was filtered and the filtrate was collected. The filtered residue was subsequently added to 750 ml ethanol solution [3/2 (v/v); ethanol/water] and the above steps were repeated. The collected filtrates were merged and filtered again. Finally, the extract was concentrated under a rotary evaporator (RE-5220, Shanghai Beilun Equipment Co., Ltd., Shanghai, China) (12).

### Cell proliferation assay

The effects of AECS on cell viability were evaluated by MTT assay. The exponentially growing A375 cells were plated in 96-well plates (Costar, Corning, NY, USA) at a density of 2×10^4^ cells/well in RPMI-1640 complete medium and following 24 h, the cells were treated with AECS at various concentrations for 24, 48 and 72 h. Fresh cell culture medium containing 10% FBS and 20 μl MTT solution (5 mg/ml) was added to each well and incubated for an additional 4 h at 37°C. Next, the medium was removed and 150 μl DMSO was added to each well. The absorbance was recorded at a wavelength of 490 nm using a microplate reader (Bio-Rad, Hercules, CA, USA) and the inhibition ratio was calculated.

### Cell cycle assay

For cell cycle analysis, A375 cells were treated with AECS at various concentrations for 48 h. Following treatment, the cells were trypsinized and fixed in ice-cold 70% ethanol overnight at 4°C, washed with phosphate-buffered saline (PBS) and stained with RNase and PI for 30 min in the dark. The cell cycle was analyzed by flow cytometry (BD FACSCalibur, Becton-Dickinson, Franklin Lakes, NJ, USA).

### Hoechst staining assay

The A375 cells were treated with various concentrations of AECS in 6-well plates for 48 h and incubated with Hoechst 33258 stain for 10 min at 37°C according to the manufacturer’s instructions. The cells were examined under a fluorescence microscope (DM505, Nikon Co., Ltd., Otawara, Tochigi, Japan).

### Flow cytometric analysis of apoptosis

The A375 cells were treated with various concentrations of AECS for 48 h, collected, washed and resuspended in PBS. The apoptotic cell death rate was examined by Annexin V-FITC and PI double staining using the Annexin V-FITC apoptosis detection kit, according to the manufacturer’s instructions. Following the staining of cells with Annexin V-FITC/PI, flow cytometry was performed and the results were analyzed using CellQuest software (BD Biosciences, Franklin Lakes, NJ, USA).

### Western blot analysis

The cells were harvested and lysed in radioimmunoprecipitation assay lysis buffer, supplemented with protease inhibitor and phosphatase inhibitor cocktail tablets. The cell lysates were centrifuged (TGL-16B, Shanghai Anting Scientific Instrument Factory, Shanghai, China) at 12,000 × g at 4°C for 10 min. Equivalent amounts of protein were subsequently resolved by 10% SDS-PAGE and transferred to polyvinylidene fluoride membranes (Millipore, Billerica, MA, USA). The membranes were blocked with Tris-buffered saline containing 0.05% Tween-20 (TBST) and 5% non-fat powdered milk for 2 h, followed by blocking with a solution, which contained the primary antibody (1:1,000 dilution) overnight at 4°C. Following three washes with TBST for 10 min, the blot was incubated with the secondary antibody (1:20,000 dilution) and washed three times with TBST prior to exposure to the SuperSignal^®^ West Dura Extended Duration substrate. The band intensity was quantified by densitometric analysis using an image quantitative analysis system (Image-Pro Plus 5.1, Media Cybernetics, Inc., Rockville, MD, USA).

### Statistical analysis

Data are presented as the mean ± standard error of the mean and statistical analysis was performed using analysis of variance. P<0.05 was considered to indicate a statistically significant difference.

## Results

### AECS suppresses A375 cell growth

**Figure 1:**
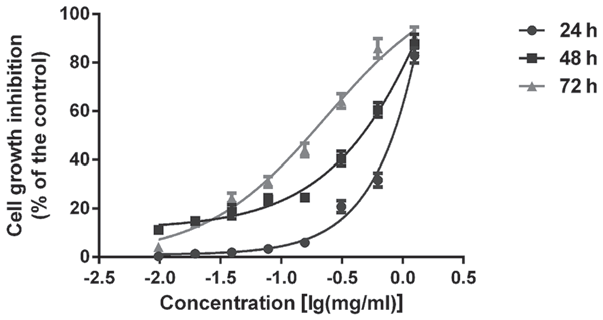
Effect of alcohol extract of centipede *Scolopendra subspinipes mutilan*s (AECS) on A375 cell growth. Cells were treated with various concentrations of AECS for 24, 48 and 72 h and cell growth was measured by 3-(4,5-dimethylthiazol-2-yl)-2,5 diphenyl tetrazolium bromide assay. Five wells were treated in each experiment and the data are presented as the mean ± standard error of the mean from three repeated experiments.

To assess the effects of AECS on cell growth, A375 cells were treated with AECS at concentrations of 0.01, 0.02, 0.04, 0.08, 0.16, 0.31, 0.63 and 1.25 mg/ml. AECS was found to inhibit the growth of A375 cells in a dose- and time-dependent manner by MTT assay (Fig. 1). In addition, the 50% growth inhibitory concentrations of AECS in A375 cells were 0.77, 0.29 and 0.15 mg/ml for 24, 48 and 72 h, respectively.

### AECS induces A375 cell S-phase arrest

**Figure 2:**
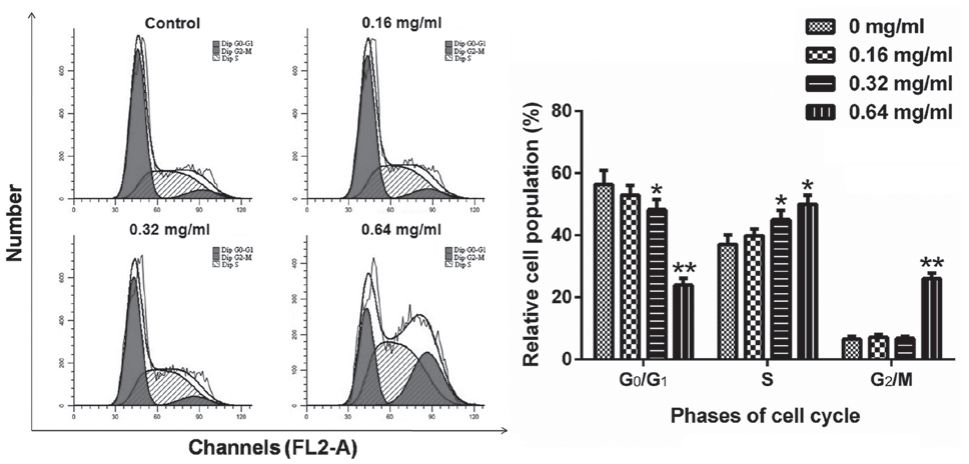
Effects of alcohol extract of centipede *Scolopendra subspinipes mutilans* (AECS) on cell cycle distribution. (A) A375 cells were treated with 0, 0.16, 0.32 and 0.64 mg/ml AECS for 48 h followed by staining with propidium iodide for flow cytometric analysis.(B) Bar charts reveal the number of cells/channel (y-axis) versus DNA content (x-axis). The values shown present the percentage of cells in the indicated phases of the cell cycle and the graph depicts the cell distribution in different phases of the cell cycle as determined by flow cytometry, which show that the treatment of A375 cells with AECS results in the blockade of cells at the S phase (Dip S). The data shown are representative of three independent experiments with similar results. Data are presented as the mean ± standard error of the mean (n=3). ^*^P<0.05 and ^**^P<0.01 vs. the control.

To further investigate the effects of AECS on the cell cycle, the cell cycle profiles of A375 cells were analyzed using flow cytometry. The cells were treated with AECS at concentrations of 0, 0.16, 0.32 and 0.64 mg/ml for 48 h and stained with PI. Next, the cells were analyzed by flow cytometry to detect their DNA content. AECS treatment resulted in a significant increase in the percentage of cells in the S phase and a significant decrease in the percentage of cells in the G0/G1 phase (Fig. 2). The percentage of cells accumulated in the S phase was 37.07, 39.85, 45.00 and 49.96% following treatment with AECS concentrations of 0, 0.16, 0.32 and 0.64 mg/ml, respectively. The accumulation of G0/G1 phase cells was maximal in the control group and declined with increasing concentrations. The decrease in the number of G0/G1 phase cells was 56.37, 52.98, 48.26 and 23.99% with AECS concentrations of 0, 0.16, 0.32 and 0.64 mg/ml, respectively. These results indicated that AECS mediates A375 cell growth by inducing partial S phase cell cycle arrest.

### Effects of AECS on cell cycle regulatory molecules

**Figure 3:**
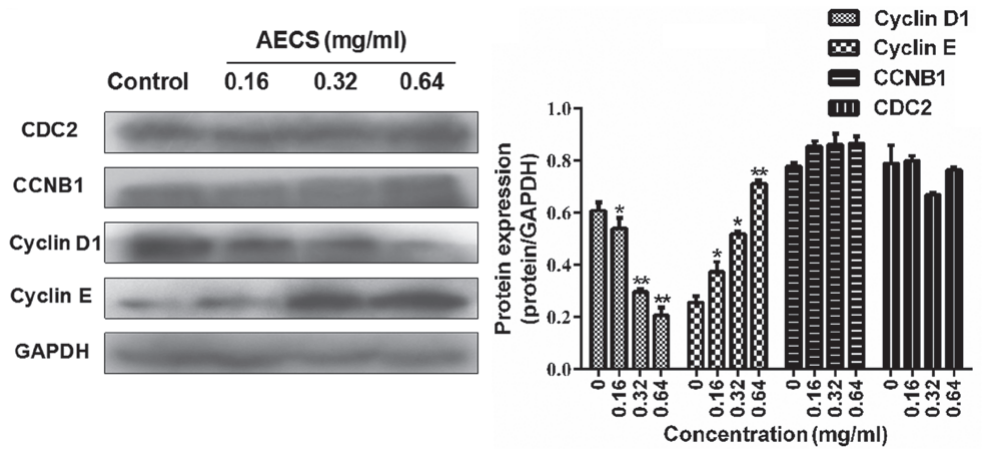
Effects of AECS on the cell cycle-related protein expression in A375 cells. Expression levels of CDC2, CCNB1, cyclin D1 and cyclin E in A375 cells treated with AECS (0, 0.16, 0.32 and 0.64 mg/ml) for 48 h were examined by western blot assay and the results were quantified by densitometry analysis of the bands and normalization to GAPDH. Data are presented as the mean ± standard error of the mean (n=3). ^*^P<0.05 and ^**^P<0.01 vs. the control. AECS, alcohol extract of centipede *Scolopendra subspinipes mutilans.*

Since AECS-induced S phase arrest was observed in the A375 cells following treatment with AECS for 48 h, the expression of cell cycle regulatory protein molecules was detected during treatment with AECS for 48 h. AECS did not affect the levels of CDC2 and CCNB1 (Fig. 3), however, treatment with AECS resulted in a subsequent increase in cyclin E expression and a significant decrease in cyclin D1 expression in a dose-dependent manner (Fig. 3). These results indicated that the cell cycle regulatory molecules are involved in AECS-induced changes in cell cycle progression.

### Effects of AECS on apoptosis

**Figure 4:**
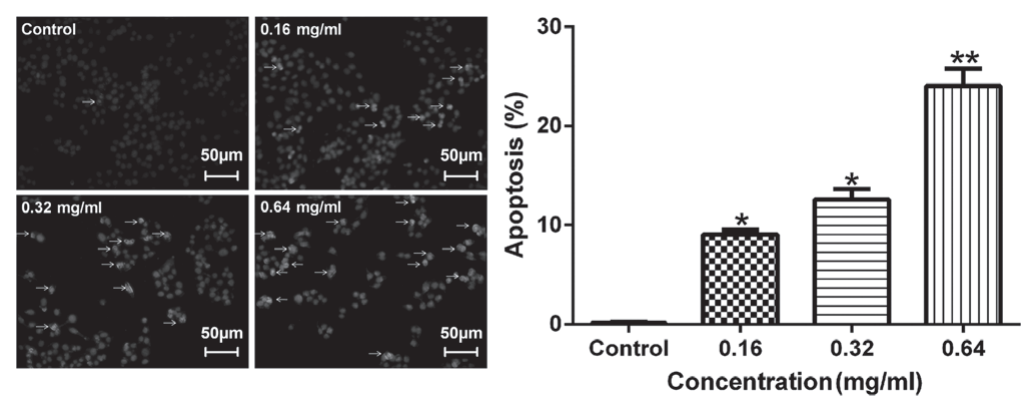
Treatment with alcohol extracts of centipede *Scolopendra subspinipes mutilans* (AECS) induces the apoptosis of A375 cells. A375 cells were cultured overnight in 6-well plates and treated in triplicate with concentrations of 0, 0.16, 0.32 and 0.64 mg/ml AECS for 48 h. AECS-induced apoptosis in A375 cells, as shown by the arrows, was characterized by nuclear condensation or nuclear fragmentation following Hoechst 33258 staining (magnification, ×200). The apoptotic cells were counted and a representative bar chart illustrating the percentage of apoptotic cells is shown. The statistically significant changes were compared and data are presented as the mean ± standard error of the mean (n=3). ^*^P<0.05 and ^**^P<0.01 vs. the control.

To detect apoptotic changes induced by AECS, the A375 cells were incubated with Hoechst 33258 dye, which is commonly used to stain genomic DNA. The Hoechst staining of the A375 cells (Fig. 4) revealed that AECS treatment induced apoptotic events characteristic of chromatin condensation. Microscopic observation (Fig. 4) demonstrated typical morphology of the apoptotic nuclei stained with Hoechst 33258, in which the chromatin was observed to be condensed and aggregated at the nuclear membrane, as indicated by a bright fluorescence at the periphery.

**Figure 5:**
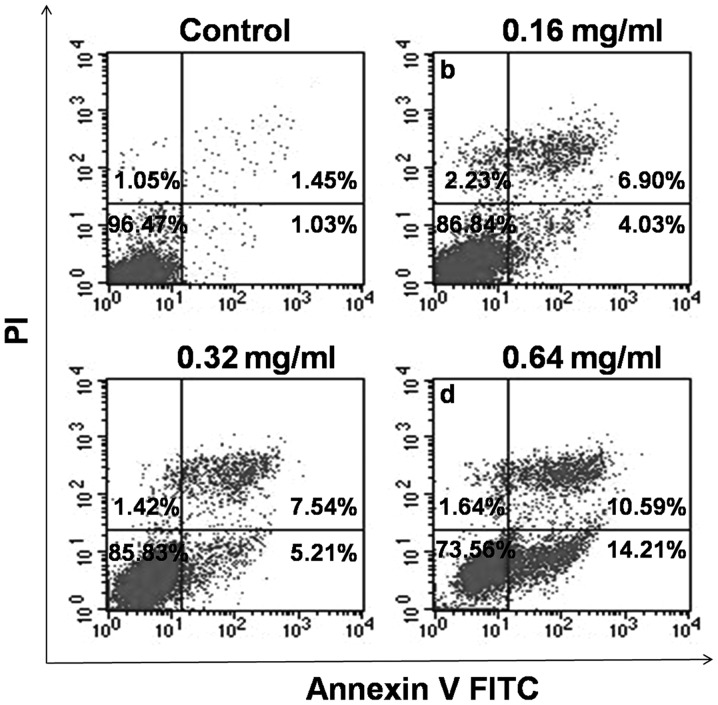
Flow cytometric analysis of AECS-induced apoptosis in A375 cells. A375 cells were cultured overnight in 6-well plates and treated in triplicate with concentrations of 0, 0.16, 0.32 and 0.64 mg/ml AECS for 48 h. The proportion of apoptotic cells was determined by double-staining with Annexin V/FITC and PI following treatment with various concentrations of AECS in A375 cells. The flow cytometry profile presents Annexin V-FITC (x-axis) and PI staining (y-axis). The values represent the percentage of cells in each of the four quadrants (lower left quadrant, viable cells; upper left quadrant, necrotic or dead cells; lower right quadrant, early-stage apoptotic cells; and upper right quadrant, late-stage apoptotic cells). Data are presented as the mean ± standard error of the mean (n=3). ^*^P<0.05 and ^**^P<0.01 vs. the control. AECS, alcohol extract of centipede *Scolopendra subspinipes mutilans*; FITC, fluorescein isothiocyanate; PI, propidium iodide.

To verify the effect of AECS on cell apoptosis, the treated cells were stained with Annexin V-FITC and PI and analyzed by flow cytometry. As shown in Fig. 5, the A375 cells treated with AECS demonstrated a significant increase in the early- and late-stage apoptotic fractions in a dose-dependent manner, which indicated that the cell growth suppression by AECS was partly due to increased apoptosis. The percentage of apoptotic cells was 2.48, 10.93, 12.75 and 24.80% in A375 cells following treatment with 0, 0.16, 0.32 and 0.64 mg/ml AECS, respectively, for 48 h.

### Effects of AECS on apoptosis regulatory molecules

**Figure 6:**
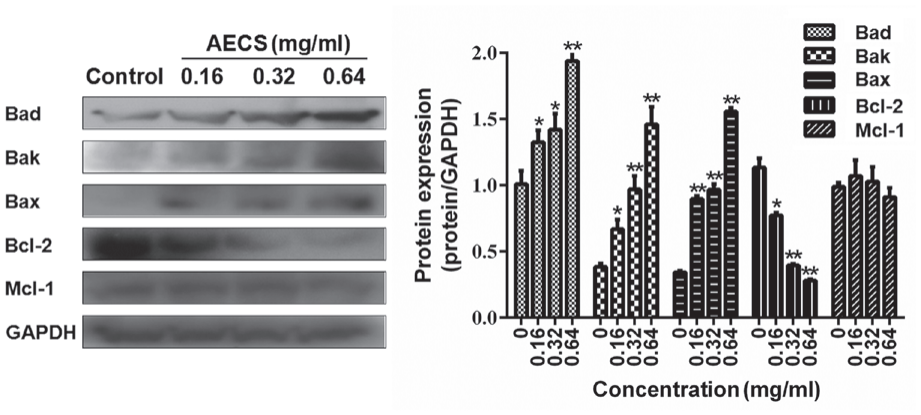
Effects of AECS on apoptosis-related protein expression in A375 cells. Expression levels of Bad, Bak, Bax, Bcl-2 and Mcl-1 in A375 cells treated with AECS (0, 0.16, 0.32 and 0.64 mg/ml) for 48 h were examined by western blot assay and the results were quantified by densitometry analysis of the bands and normalization to GAPDH. Data are presented as the mean ± standard error of the mean (n=3). ^*^P<0.05 and ^**^P<0.01 vs. the control. AECS, alcohol extract of centipede *Scolopendra subspinipes mutilans.*

It was hypothesized that the AECS-induced apoptosis observed may result from the effect on the Bcl-2 family members. Therefore, the expression of the Bad, Bak, Bax, Bcl-2 and Mcl-1 proteins in the A375 cells treated with increasing concentrations of AECS for 48 h were investigated by western blot analysis. AECS did not affect the expression levels of Mcl-1 (Fig. 6), however, treatment with AECS significantly decreased Bcl-2 expression and increased Bad, Bak and Bax expression in a dose-dependent manner in the A375 cells (Fig. 6).

## Discussion

In the present study, it was demonstrated that AECS was involved in inhibiting A375 cell proliferation via the induction of cell cycle arrest and apoptosis. In addition, the effect of AECS on A375 cell growth was investigated using an MTT assay. The results revealed that AECS exhibits a significant inhibition of A375 cell growth in a dose- and time-dependent manner. As cell cycle arrest and apoptosis represent two effective mechanisms involved in the induction of cell death (13), further investigation of the effect of AECS on cell cycle arrest and apoptosis was performed in the present study, in addition, the associated underlying molecular mechanism of AECS-induced cell cycle arrest and apoptosis in A375 cells was investigated.

Eukaryotic cell proliferation is primarily regulated by the cell cycle, which consists of four phases: The S phase, DNA synthesis phase; the M phase, mitosis; the G1 phase, prophase DNA synthesis; and the G2 phase, anaphase DNA synthesis (14). Furthermore, it is well established that the loss of key cell cycle checkpoints is a hallmark of cancer cells, which leads to abnormal proliferation and facilitates oncogenic transformation (15). The G1/S transition is one of the two predominant checkpoints of the cell cycle, and is responsible for the initiation and completion of DNA replication. The majority of studies have reported perturbation of the S/G2 phase transition with a decrease of cells in the G0/G1 phase of the cell cycle and an increase of cells in the S phase (15,16). In the present study, FACS analysis with PI staining revealed that the percentage proportion was increased in the S phase cells and reduced in the G0/G1 phase cells following AECS treatment in a dose-dependent manner, indicating that the inhibitory effect of AECS on A375 cell proliferation is mediated by S phase cell cycle arrest.

Progression through the cell cycle is regulated by the coordinated action of cdks and their associated regulatory subunits, cyclins. In addition, studies have demonstrated that progression through the G1/S transition is regulated by cyclin E (17), which is expressed in late G1, preceding cyclin A expression, with maximal expression observed at the G1/S boundary. Cyclin E-cdk2 activity is maximal near the G1/S boundary and is required for the G1 to S phase transition (18). In the current study, the expression of these important regulatory proteins was analyzed following the treatment of A375 cells with AECS and the results were consistent with previous observations that S phase arrest is accompanied by the increased expression of cyclin E (19) and the decreased expression of cyclin D1. The modifications of these cell cycle-associated proteins induced by AECS appear to perturb the cell progression through the S phase.

Clear evidence exists that tumor growth is a result of uncontrolled proliferation and reduced apoptosis, thus, inducing cancer cell apoptosis is a key strategy in anticancer therapy (20). Inducing apoptosis contributes to cancer treatment through various mechanisms, including preventing growth-factor-independent cell survival, inhibiting resistance to immune-based cytotoxicity and interfering with the bypassing of cell cycle checkpoints (11). Thus, the current study performed flow cytometric and Hoechst 33258 staining assays to observe the apoptotic effects of AECS on A375 cells. It was revealed that AECS treatment induces apoptotic events, which are characteristic of chromatin condensation and significantly increase the apoptotic fraction of A375 cells in a dose-dependent manner; this indicated that the inhibitory effect on tumor cell proliferation by AECS was partially due to the effect of inducing apoptosis.

To gain further insight into the mechanism of AECS-induced cell apoptosis, its effect on the protein levels of the Bcl-2 family was determined by western blot analysis. The members of the Bcl-2 family have an important function in the regulation of cell survival/apoptosis by serving as antiapoptotic (for example Bcl-2 and Bcl-xl) or proapoptotic (such as Bax and Bad) proteins. The balance between these two classes of proteins is critical for determining whether a cell undergoes apoptosis (21). Therefore, the current study detected the protein expression of Bcl-2, Mcl-1, Bak, Bax and Bad in A375 cells using western blot analysis, which revealed that AECS induces the downregulation of Bcl-2 expression, correlating with the upregulation of Bak, Bax and Bad expression. The results showed that the apoptosis-inducing effect of AECS in A375 cells is significantly associated with the effects on the Bcl-2 family and that A375 cell apoptosis by AECS contributes to the inhibition of cell growth.

In conclusion, the present study demonstrated that AECS inhibits the growth of A375 cells by arresting the cell cycle at the S phase and inducing cell apoptosis. Therefore, the use of AECS may present a potential strategy for the treatment of human melanoma cancer.
